# A Long-Range Distributed Antenna Method Based on Microwave-Photonics Frequency Synchronization

**DOI:** 10.3390/e28091024

**Published:** 2026-09-14

**Authors:** Haotian Teng, Mingtuan Lin, Hui Han, Hao Gao, Xinglin Qin, Baichi Chen, Yuanmei Xie, Juntao He, Bo Liu

**Affiliations:** 1 Department of Advanced Interdisciplinary Sciences, National University of Defense Technology, Changsha 410073, China; tenghaotian19@nudt.edu.cn (H.T.); hejuntao12@163.com (J.H.); 2 Department of Electronic Science and Technology, National University of Defense Technology, Changsha 410073, China; linmingtuan08@163.com (M.L.); sdqxl123@163.com (X.Q.); 19967903125@163.com (B.C.); 3 Department of Computer Science, National University of Defense Technology, Changsha 410073, China; hanhui@nudt.edu.cn; 4 State Key Laboratory of Information Photonics and Optical Communications, Beijing University of Posts and Telecommunications, Beijing 100876, China; hao@bupt.edu.cn; 5 College of Science, National University of Defense Technology, Changsha 410073, China; xieyuanmei@nudt.edu.cn

**Keywords:** frequency synchronization, microwave photonics, distributed antenna, phase-locked loop, coherent beamforming

## Abstract

Coherent signal synthesis among multiple remote apertures is a fundamental challenge for next-generation distributed radar and communication systems, and its performance hinges on the precision of the frequency and time references shared by the distributed nodes. In this work, we report a system-level study of a microwave-photonics-based frequency synchronization system that phase-locks two independent signal sources over a 40 km fiber link, and we validate the resulting coherent beamforming and power combining capabilities of the synchronized pair. The synchronization link, built on an adaptive phase-locked loop with dispersion compensation, delivers a frequency stability of $8.0\times 10^{-15}$ at 1 s and $2.0\times 10^{-16}$ at 1000 s. Using the synchronized pair, we perform beam-scanning and beamforming experiments in a microwave anechoic chamber at 1–7 GHz. The measured beam-pointing angles agree with theoretical predictions, and a coherent gain enhancement of 5.9 dB is obtained with a gain loss below 0.1 dB. Furthermore, field tests with a 150 m free-space separation between the two transmitting antennas confirm stable coherent signal synthesis, with the combined amplitude maintained within 1 dB over extended periods. Beyond the specific experimental results, we apply the known gain-loss relation to derive an engineering guideline relating the frequency stability of the synchronization link to the achievable coherent gain loss. These results show that an established microwave-photonics synchronization technology, when integrated with standard signal sources, provides a practical route toward distributed coherent arrays for long-range detection and wideband communication.

## 1. Introduction

**Figure 1:**
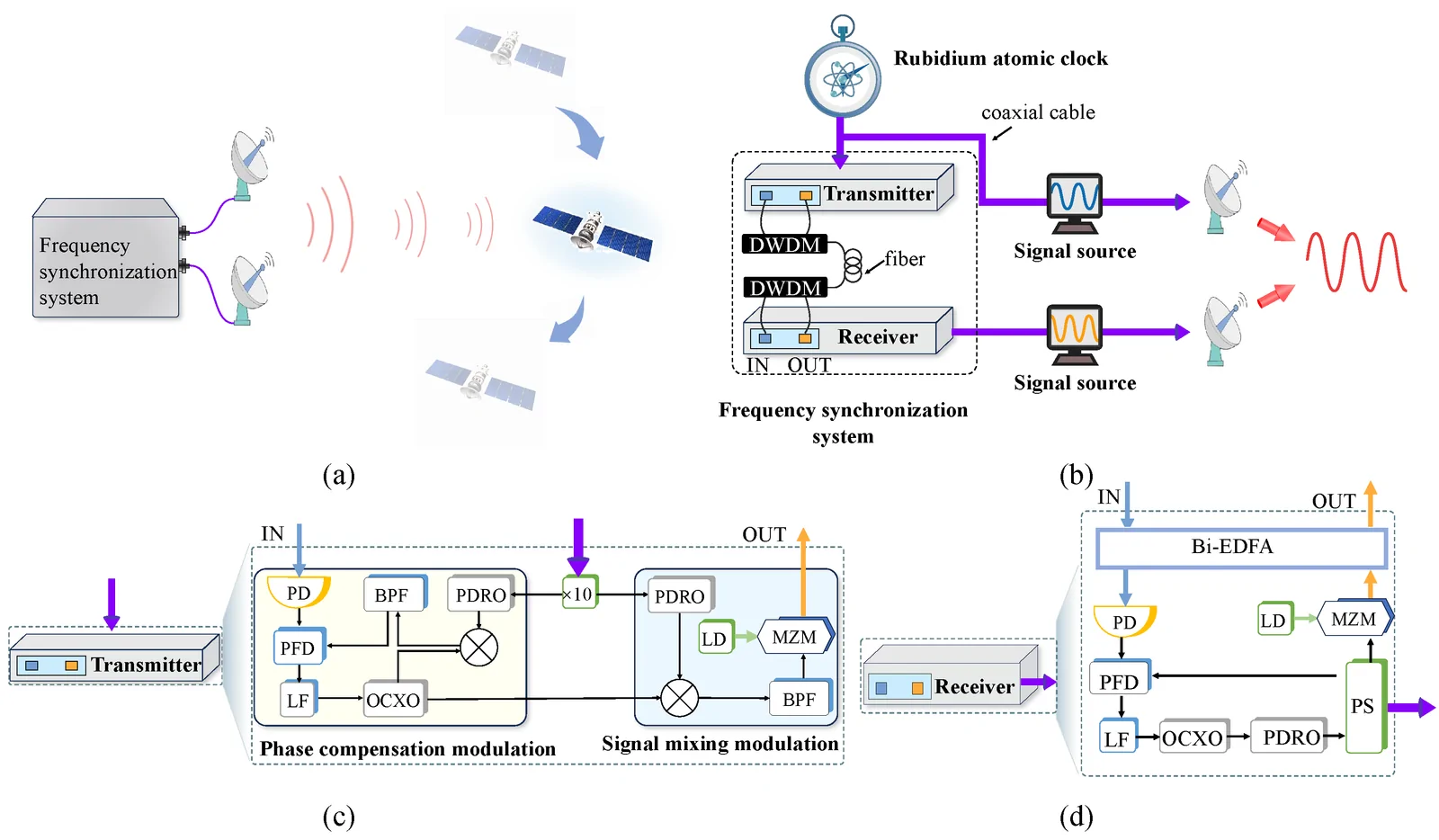
Distributed antenna system based on microwave-photonic frequency synchronization technology. (**a**) Distributed coherent radar application scenario. (**b**) Distributed antenna architecture based on microwave-photonic frequency synchronization. (**c**) Device structure of the transmitter modules. (**d**) Device structure of the receiver modules. PD: photodetector; DWDM: dense wavelength-division multiplexing; PDRO: phase-locked dielectric resonant oscillator; OCXO: oven-controlled crystal oscillator; BPF: band-pass filter; LF: loop filter; PFD: phase-frequency detector; LD: laser diode; MZM: Mach–Zehnder modulator; PS: power splitter; Bi-EDFA: bidirectional erbium-doped fiber amplifier.

Coherent signal synthesis among multiple spatially separated apertures is a fundamental enabler for next-generation distributed radar and communication systems. As communication demands grow, system effectiveness is influenced by factors like the power–aperture product, signal-to-noise ratio (SNR), and bandwidth. While traditional large-aperture wideband antennas can boost signal strength, they often suffer from limited mobility and high deployment costs [1]. Where single-node systems become ineffective, by distributing multiple antenna elements spatially, these systems achieve coherent signal synthesis that effectively enhances the SNR and extends communication range [2]. This technical concept has been naturally extended to the radar diagram, giving rise to the distributed coherent aperture radar (DCAR) [3], as shown in Figure 1a. Two operation modes are commonly distinguished in such systems: coherent-on-receive, where only the received signals of the *N* apertures are combined and the SNR gain scales approximately as *N*, and fully coherent operation, where both transmit and receive apertures are phase-aligned and the SNR gain scales as $N^{3}$ [4,5,6]. Under fully coherent transmit and receive conditions, a distributed antenna network with *N* units can thus achieve an $N^{3}$ times SNR gain, enabling reliable long-range communication and detection that would be unachievable with conventional single-platform solutions.

Early studies pioneered full-coherence field experiments based on distributed coherent radar configurations, at a specific frequency, successfully achieving synthetic gains of approximately 6 dB for reception coherence and nearly 9 dB for full coherence [6]. However, these initial efforts lacked targeted optimization of frequency synchronization accuracy, leaving room for improvement in the stability of coherent synthesis. More recently, a photonics-based coherent multiple-input multiple-output (MIMO) distributed radar network was demonstrated in field trials, in which two radar heads operating in different frequency bands were interconnected through fiber and their received signals were combined coherently at a central unit [7]; the coherence of such systems, however, still relies on the stability of the deployed fiber links rather than on an actively compensated frequency reference. Further work carried out principle verification experiments on an X-band two-unit distributed coherent microwave photonic radar synchronized through a 12 km fiber-optic time and frequency network, achieving SNR gains of about 8.1 dB and 7.9 dB under full coherence [8]. Nonetheless, the demonstrated time synchronization accuracy of approximately 26 ps still depends on the stability of the transmission links, and long-range or complex-environment operation remains to be validated. An alternative signal-processing-based route compensates the time, frequency, and phase mismatches through dominant-scatterer estimation, realizing coherent-on-receive synthesis in millimeter-wave DCAR without a dedicated synchronization link [9]; this approach, however, cannot support fully coherent transmit operation, and therefore leaves the frequency synchronization of spatially separated sources as an open issue for coherent synthesis.

Effective signal phase-coherent synthesis requires high-precision frequency synchronization [10,11], with atomic clocks serving as the core benchmark for precise time–frequency systems due to their excellent long-term stability [10,11,12,13,14]. However, the reliable remote transmission of high-precision signals remains a critical challenge. While satellite-based frequency transmission offers virtually unlimited transmission distance, it is plagued by propagation noise from ionospheric effects, tropospheric disturbances, and multipath interference, which significantly degrade the received frequency reference signal [15]. Wireless synchronization schemes, utilizing dual-tone signaling, custom-filtered reference clock extraction, and dynamic frequency selection, can achieve a time precision of 5 ns and a frequency precision of 0.1 Hz, comparable to wired solutions [16,17,18,19,20]. However, challenges such as wide beam spread and multipath interference hinder their ability to meet the high-precision requirements of modern distributed systems. In contrast, optical signal transmission offers advantages such as low loss, strong immunity to electromagnetic interference, and ultra-wide bandwidth, making it ideal for converting electrical signals into optical signals for long-distance transmission. Nevertheless, the real-world deployment of optical fibers remains affected by environmental factors such as mechanical vibrations and temperature fluctuations, which degrade the stability of transmitted frequency signals [7,21,22,23]. Therefore, developing advanced detection and compensation techniques is essential to enhance the robustness of fiber-based frequency transfer [24,25].

We note that fiber-based frequency transfer has a history of nearly two decades, with optical-frequency dissemination over deployed links reaching fractional stabilities of $2\times 10^{-19}$ at 30,000 s over 540 km [26]. However, such optical-frequency transfer primarily targets optical-clock comparison and relies on ultrastable lasers with active noise compensation. Distributed radar and communication systems, in contrast, require the dissemination of a microwave reference that directly drives electronic signal sources; for this microwave domain, White-Rabbit-based time and frequency transfer [27] and Global Positioning System (GPS)-disciplined oscillator synchronization [28] represent the practical baseline. Between these two domains, optical frequency combs provide a dense grid of phase-coherent tones traceable to the atomic reference and allow many frequency channels to be disseminated over a single fiber. Our work focuses on this microwave domain and on the integration of an established microwave-photonics synchronization system with standard signal sources for coherent synthesis.

In this work, we report a system-level study of a microwave-photonics-based frequency synchronization system that phase-locks two remotely located signal sources, and we validate the resulting coherent beamforming and power combining for distributed antenna applications. The motivation is practical: in distributed coherent radar and communication systems, the $N^{3}$ SNR gain promised by fully coherent operation of *N* apertures is reachable only if the frequency references at the distributed apertures are mutually stable at the level that keeps the coherent gain loss below the acceptable threshold; this requirement, quantified by Equations (2) and (3) as a $10^{-14}$ stability threshold in Section 2.2, becomes the design target that the synchronization subsystem must meet. Our contribution is threefold, and each contribution is supported by a dedicated experiment, so that the three experiments form a closed validation chain. First, we demonstrate that a synchronization link built on an adaptive phase-locked loop with dispersion compensation can sustain a frequency stability of $8.0\times 10^{-15}$ at 1 s and $2.0\times 10^{-16}$ at 1000 s over a 40 km fiber link, the latter exceeding the $10^{-14}$ coherence requirement derived in Section 2.2 by a factor of 50; this capability is certified by the long-haul link experiment of Section 4.1, which also validates the engineering guideline relating the frequency stability of the synchronization link to the achievable coherent gain loss. Second, with the reference stability certified, we show that the synchronized pair supports broadband coherent operation: beam-scanning and beamforming across the 1–7 GHz band in a microwave anechoic chamber, with beam-pointing angles agreeing with the array theory (Section 4.2), and stable coherent signal synthesis over a 150 m free-space separation in a field test (Section 4.3), achieving a coherent gain enhancement of 5.9 dB with a gain loss below 0.1 dB, close to the 6 dB theoretical limit of dual-aperture synthesis. Third, these results translate into a concrete deployment position: the demonstrated architecture is compared against GPS-disciplined, White-Rabbit-based, and wireless synchronization alternatives, and we argue that microwave-photonics synchronization is preferable in Global Navigation Satellite System (GNSS)-denied or reconfigurable scenarios, where a fiber-connected, source-independent frequency reference is required. These results show that an established microwave-photonics synchronization technology, integrated with standard signal sources, can effectively coordinate distributed signal sources and provide a practical path toward distributed coherent arrays for long-range detection and wideband communication.

## 2. Frequency Synchronization System and Coherence-Requirement Analysis

### 2.1. System Architecture and Operating Principle

Before describing the hardware in detail, we briefly summarize the operating principle of the synchronization system, which follows the phase-stabilized frequency-transfer scheme of Refs. [24,29]. Two categories of noise accumulate over a long fiber link: (i) amplified spontaneous emission (ASE) noise from the bidirectional erbium-doped fiber amplifiers, which appears as additive amplitude noise on the detected microwave signal after photodetection, and (ii) coherent noise arising from residual chromatic dispersion, which converts laser phase noise into phase fluctuations in the detected signal. The system suppresses both through a closed loop: the phase-frequency detector compares the round-trip signal with the local reference, and the loop filter drives an oven-controlled crystal oscillator so that phase fluctuations within the loop bandwidth are tracked while those outside the bandwidth are rejected. A dual-tone architecture realizes phase-conjugate pre-compensation: the phase-locked dielectric resonant oscillator generates two tones, which are mixed with the OCXO output and loaded onto the optical carrier; the round-trip signal carries the link-phase fluctuation, and the Transmitter impresses the inverse of this fluctuation onto the forward signal, so that the link noise is cancelled upon transmission. As analyzed in Ref. [29], the loop bandwidth must balance the suppression of the ASE-induced amplitude noise against the dispersion-converted phase noise, rather than being set by the transmission delay alone.

The detailed architecture of the distributed antenna system is illustrated in Figure 1b, consisting mainly of three major components: a rubidium atomic clock, a frequency synchronization system, and two signal sources with amplitude phase control. The atomic clock serves as the core frequency reference source for the entire system, providing a 10 MHz reference clock signal through a clock distribution network connected to the frequency synchronization system. This frequency synchronization system employs a microwave-photonics-based phase-locking technology, ensuring high consistency of the phase and amplitude of the reference signal during long-distance transmission through precisely designed frequency conversion, transmission, and down-conversion processing. Inside the two signal sources, amplitude phase-control units adjust the output phase of the signals so that their coherent superposition in the target area is maximized. Combined with the principle of beamforming, adjusting the phase difference of each array element signal achieves coherent superposition of signals in the target area, thereby maximizing signal synthesis intensity.

To be more specific, Figure 1c,d shows the device structure of the transmitter and receiver modules. The transmitter modules use a rubidium atomic clock as the core frequency source, which drives the phase-locked dielectric resonant oscillator (PDRO) at the transmitting end to generate dual-frequency microwave signals. In the phase compensation modulation module, an internal phase-locked loop (PLL) processes the returned signal from the receiver, which carries link noise information, and aligns it in frequency and phase with a reference signal generated by a band-pass filter. The aligned signal is then fed back to the signal mixing modulation. A compensation component will be imparted with the opposite phase to the link noise onto the signal from the oven-controlled crystal oscillator (OCXO) before it enters the signal mixing module. In this way, after the signal generated by the OCXO is output to the signal mixing modulation, the signal will carry the link noise with the inverted phase. The signal mixing modulation mixes the dual-frequency signals output by the PDRO with the signals output by the OCXO to generate transfer signals. The PDRO generates two microwave tones at the Transmitter: $f_{1}$ serves as the reference to be disseminated, while $f_{2}$ serves as a probe tone for link-noise cancellation. After photodetection and mixing at both ends of the link, the round-trip comparison cancels the common-mode link noise, so that the receiver can reproduce the atomic-clock reference. The specific frequencies used in the experiment are listed in Table 1. These signals are transmitted through several tens of kilometers of optical fiber, leveraging the optical transmission mechanism to suppress electromagnetic interference while carrying link information. After the signal reaches the receiver end, through the action of the phase-locked loop, one path serves as the output signal interface, and the other path is transmitted back to the phase compensation modulation module at the transmitter end through the same optical fiber link. The forward and looped-back signals thus propagate in opposite directions over a single bidirectional standard single-mode fiber; this topology is essential for the round-trip cancellation of link noise. This cancels out the link noise, enabling the receiver to generate a 10 MHz signal that remains highly consistent with the atomic-clock reference in both frequency and phase.

Dispersion compensation is a critical enabling technique for stable radio frequency (RF) transmission over spans of hundreds of kilometers. In this work, a hardware-level scheme is adopted where each bidirectional erbium-doped fiber amplifier (Bi-EDFA) module incorporates a segment of dispersion-compensating fiber (DCF) designed to cancel the dispersion accumulated in approximately 80 km of standard single-mode fiber (SMF). These DCF-equipped amplifiers are inserted along the entire link, preserving pulse fidelity and reducing the signal degradation experienced by the subsequent phase-locked loop (PLL). This approach tightly couples dispersion management with optical amplification, and the total DCF loss budget must be carefully accounted for in the link power design.

While hardware compensation brings the residual dispersion down to a low level, the remaining chromatic dispersion can still translate into phase noise that limits the system’s ultimate frequency stability. In [29], the residual dispersion coefficient ψ is explicitly modeled as

(1) $$\psi =2\pi \lambda \left(\sum_{i=1}^{N}D_{i}\cdot l_{i}\right),$$

where λ is the operating wavelength, and $D_{i}$ and $l_{i}$ are the dispersion coefficient and length of the *i*-th fiber segment, respectively. This accumulated residual dispersion converts laser phase noise into intensity noise, which then enters the PLL feedback path. The key insight of Ref. [29] is that the PLL bandwidth should not be chosen simply from the transmission delay; instead, there exists an optimal bandwidth that balances the suppression of EDFA-induced intensity noise against the dispersion-converted phase noise. With such an optimized PLL, the long-term stability improves markedly, demonstrating that an integrated hardware-plus-system strategy—where the residual dispersion is mathematically modeled and its noise contribution is actively mitigated by loop tuning—is essential for pushing frequency dissemination toward the ultimate stability limit.

### 2.2. Frequency-Stability Requirement for Coherent Synthesis

A direct consequence of imperfect synchronization in distributed systems is the presence of time and phase deviations in the transmit and receive signals of each aperture. The synthesized signal with frequency synchronization deviation and the resulting synthetic gain loss (in dB) under ideal conditions are expressed as follows [4]:

(2) $$A\left(t\right)=\sum_{n=1}^{N}e^{j\epsilon_{\Psi_{n}}};$$

(3) $$G_{\mathrm{loss}}=-10\log_{10}\left(\frac{1}{N^{2}}\left|A\left(t\right)A^{*}\left(t\right)\right|\right).$$

Here, *N* denotes the number of apertures; $\epsilon_{\Psi_{n}}=\pi Tf_{c}\delta f_{\mathrm{ref},n}$ is the instantaneous phase deviation induced by frequency drift, where *T* is the total observation time; $f_{c}$ is the signal carrier frequency; and $\delta f_{\mathrm{ref},n}$ represents the frequency stability of the *n*-th aperture. Note that in Equations (2) and (3), we assume zero time deviation and zero fixed phase offset, as this work focuses on the impact of frequency synchronization on coherent synthesis. Under ideal conditions, a gain loss of $G_{\mathrm{loss}}<0.5\;\mathrm{dB}$ is generally considered acceptable, since this threshold satisfies the mutual coherent-integration requirement between distributed antenna elements and prevents significant degradation in coherent synthesis performance.

**Figure 2:**
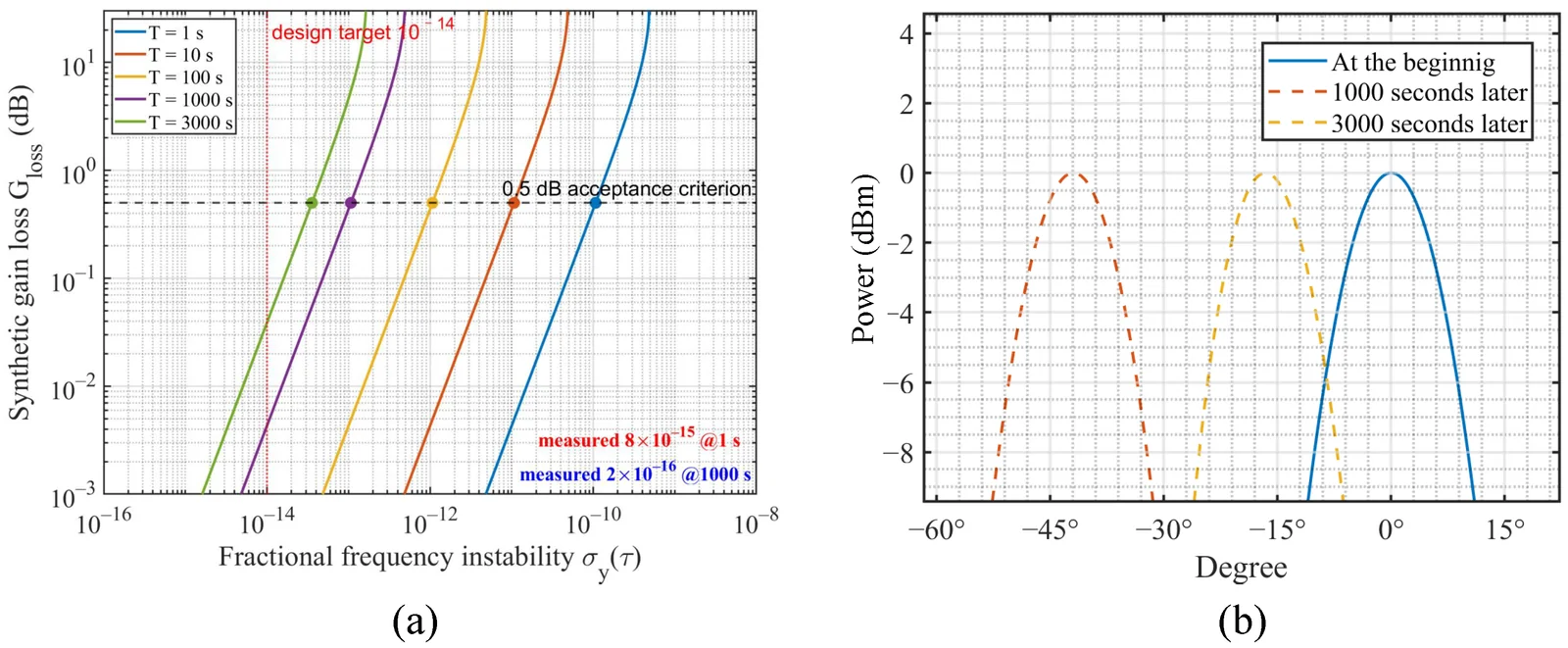
Impact of frequency stability on coherent synthesis (simulation). (**a**) Synthetic gain loss versus fractional frequency instability for 2 GHz signals under observation windows of 1, 10, 100, 1000 and 3000 s; the dashed line marks the 0.5 dB acceptance criterion, the dotted line marks the $10^{-14}$ design target, and the two markers indicate the measured stability of the synchronization link (Section 4.1). (**b**) Beamforming pattern of the dual-antenna system under different frequency instabilities.

Based on Equation (2) and (3), Figure 2a evaluates the synthetic gain loss of a dual-aperture system versus the fractional frequency instability of the references. Following the phase-error model of Ref. [4] (Equation (6) therein), the frequency offset of each aperture is assumed to grow linearly from zero to its Allan-deviation value within the observation window, so that the cumulative phase deviation over a window *T* is $\Delta \phi =\pi f_{c}\sigma_{y}T$; taking the two apertures as independent, the relative phase deviation is conservatively bounded by the same expression. With the 0.5 dB acceptance criterion stated above, the simulation sets the design target of the frequency stability at $10^{-14}$ for a 1000 s observation window at 2 GHz, which corresponds to a cumulative phase deviation of only 3.6° and hence a predicted gain loss of 0.004 dB; the measured stability of the synchronization link ($8\times 10^{-15}$ at 1 s and $2\times 10^{-16}$ at 1000 s, Section 4.1) stays well below this target, providing a comfortable margin for coherent operation. Insufficient synchronization also makes the beam roll: a residual frequency offset Δf between the two sources sweeps the array phase at 2πΔf rad/s, so the main beam periodically rolls across the scan range with period 1/Δf while the gain at broadside oscillates. To quantify the impact of frequency stability on beamforming, Figure 2b plots the beamforming patterns of the dual-antenna system under different frequency instabilities. As time accumulates, the relative phase drift shifts the beam-pointing direction, which follows $\theta_{0}\left(T\right)=\arcsin\left(f_{c}\;\sigma_{y}\;T\;\lambda /\left(2d\right)\right)$, with d=43.5 cm being the antenna spacing: with a frequency instability of $6.5\times 10^{-13}$ at 2 GHz, the main lobe is deflected by about 13° at 1000 s and by more than 40° at 3000 s, matching the pattern shift shown in Figure 2b; in contrast, at the design target of $10^{-14}$, the cumulative deflection remains below 0.6° even after 3000 s, which is negligible. This shift indicates that insufficient frequency stability leads to cumulative phase drift, which increases with time—even a small deviation in frequency stability will result in significant phase errors over extended periods. The measured stability of the actual synchronization link is presented in Section 4.1, where it is compared against this simulation-derived requirement.

## 3. Distributed Theoretical Analysis

Based on the frequency synchronization system, to further clarify the coherent synthesis and beam-scanning mechanism of distributed antennas, theoretical analysis is conducted.

For dual-antenna systems, when two signals have equal amplitude, precise phase adjustment of one signal coordinates the phase relationship between the two, maximizing the synthesis effect and achieving peak gain, so the received signal strength can be significantly increased. To quantitatively analyze how time difference, frequency difference, and phase difference affect coherent synthesis, the complex forms of two signals in a dual-antenna system can be defined as follows:

(4) $$\begin{array}{cc} s_{1}\left(t\right) & =A_{1}e^{j\left[2\pi \left(-f_{L}\right)t+\phi_{1}\right]},\\ s_{2}\left(t\right) & =A_{2}e^{j\left[2\pi \left(-f_{L}+f_{d}\right)\left(t-t_{i}\right)+\phi_{2}\right]}, \end{array}$$

where $A_{1}$ and $A_{2}$ denote the amplitudes of the two signals, respectively; $f_{L}$ is the local oscillation frequency of both signals; $f_{d}$ represents the frequency difference between the two signals; $t_{i}$ is the time delay difference between the two signals; and $\phi_{1}$ and $\phi_{2}$ are the initial phases of the two signals, respectively.

The time delay difference between antenna signals originates from discrepancies in signal arrival time and transmission delay. Since the received signal only carries time, frequency, and phase difference information from the target source, uncompensated differences will severely limit the SNR improvement of coherent synthesis algorithms. To resolve this, it is necessary to first compensate for the signal time difference $t_{i}$ and frequency difference $f_{d}$. After compensation, the intensity of the synthesized signal $I_{0}$ can be expressed as:

(5) $$I_{0}=I_{1}+I_{2}+2\sqrt{I_{1}I_{2}}\cos\left(\Delta \phi \right),$$

where $I_{1}$, $I_{2}$ is single signal strength, and Δϕ is the difference value of $\phi_{1}$ and $\phi_{2}$. From this formula, when phases are consistent, Δϕ=0, and the received signal strength is 6 dB higher than the sum of individual signal strengths, which quantifies the theoretical gain of coherent synthesis and provides a benchmark for subsequent experiments.

To guide practical phase adjustment for beam-scanning, we derive the phase difference formula for array elements. For the azimuth-plane beamforming used in our experiments, the phase adjustment value of each array element is determined by the target beam-pointing angle:

(6) $$\Delta \phi =\frac{2\pi d}{\lambda }\sin\left(\theta \right).$$

Combining Equations (5) and (6), the synthetic gain loss follows $G_{\mathrm{loss}}\left(\Delta \phi \right)=-20\log_{10}\left[\cos(\Delta \phi /2)\right]$: for phase differences of 0°, 30°, 60°, 90°, and 120°, the loss is 0, 0.30, 1.25, 3.01, and 6.02 dB, respectively. Two conclusions follow. First, the peak gain at the steered direction depends only on the inter-element phase difference; the steering angle only determines the required phase shift through Equation (6). Second, what changes with the steering angle is the pattern geometry rather than the peak gain: when the phase difference between the two signal sources is adjusted, the azimuth at which the maximum gain occurs shifts accordingly. According to this formula, a phased array driver adjusts the phase of each element’s signal to form a directional beam, enabling beam scanning—gradual adjustment of the phase difference shifts the beam from one angle to another. For applications requiring real-time target tracking, such as radar and satellite communication, the system dynamically adjusts the phase based on the target trajectory and implements feedback control to maintain beam alignment.

## 4. Experiments and Results

### 4.1. Experiment on the Long-Haul Synchronization Link

As the first layer of the three-layer validation outlined in the Introduction, this experiment verifies our first contribution: whether the synchronization link can deliver the frequency stability required by the coherence condition derived in Section 2.2. To this end, the frequency stability of the microwave-photonics synchronization system over a 40 km fiber link was characterized by a dedicated stability test.

**Figure 3:**
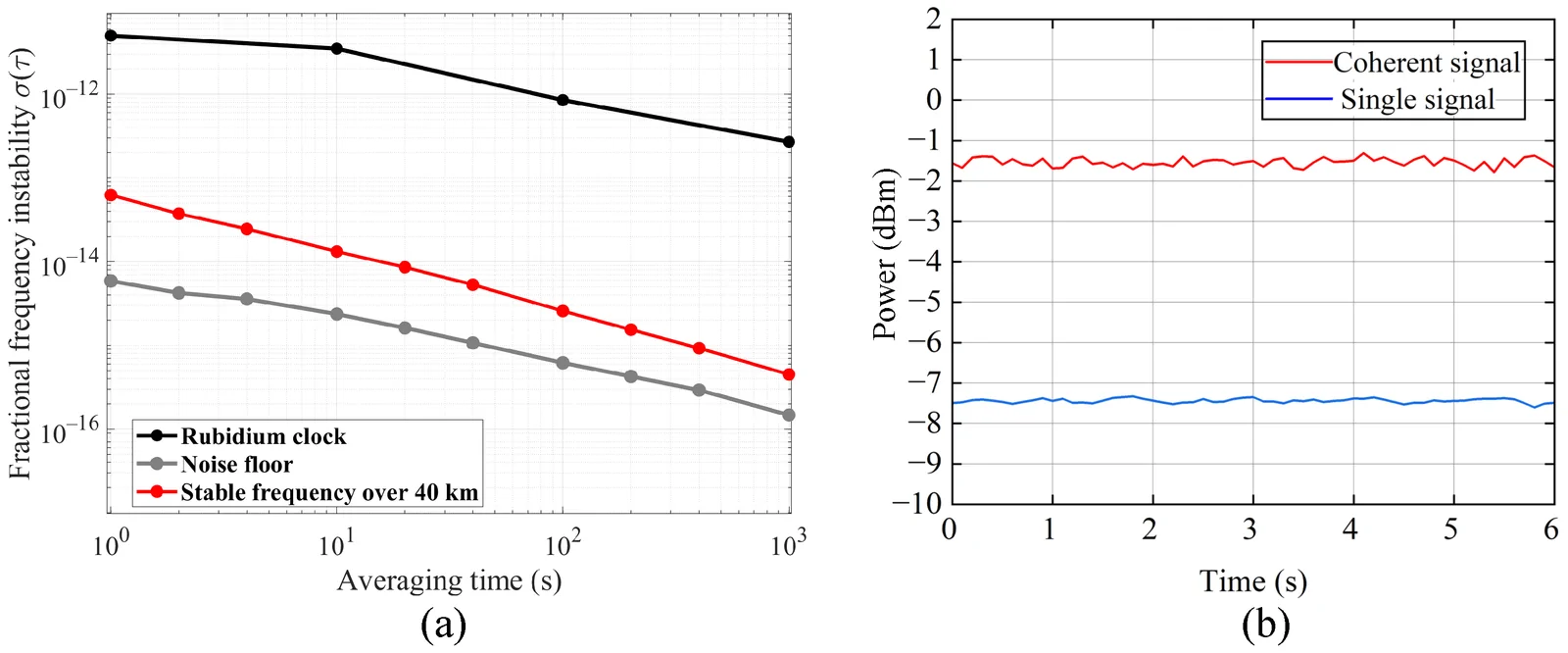
(**a**) Frequency stability of different transmission routes: the atomic-clock reference, the frequency synchronization system, and the measurement noise floor. (**b**) Amplitude stability of the synthesized signal when the two sources are locked by the synchronization system.

The results are shown in Figure 3a. Three curves are presented: the frequency stability achieved via atomic-clock connection, the frequency stability after adopting the high-precision frequency synchronization system, and the frequency stability under the noise floor (i.e., the frequency stability when devices are directly connected, considering only noise interference excluding that from the transmission link). Notably, the frequency stability of the frequency synchronization system is very close to the noise floor. The system exhibits excellent frequency stability, achieving $8\times 10^{-15}/s$ and $2\times 10^{-16}/1000\;s$.

To investigate the impact of synchronization quality on signal amplitude, a signal-source synchronization comparison experiment was conducted. When the microwave-photonics-based PLL is applied to synchronize the two sources, the amplitude performance is shown in Figure 3b. In this case, the synthesized signal becomes highly stable. The adaptive PLL, together with dispersion compensation, suppresses the relative phase drift between the two sources to below $10^{-4}$ rad/s (equivalent to the measured $8\times 10^{-15}$ fractional frequency deviation at 2 GHz). Once the inter-signal phase difference is stabilized, the synthesized amplitude remains constant and increases by 5.9 dB, clearly demonstrating the high stability of the synchronization system.

Taken together, the experimental results highlight the critical influence of frequency stability on coherent beamforming performance and confirm the effectiveness of the employed microwave-photonics-based phase-locking system. This experiment directly supports the first contribution: the measured stability at 1000 s exceeds the $10^{-14}$ requirement derived from the coherence condition by a factor of 50, providing a comfortable margin for coherent operation at C-band and higher frequencies.

### 4.2. Experiment in Microwave Anechoic Chamber

To verify the distributed antenna system’s beam control capability and phase synchronization accuracy, we performed beamforming experiments in a microwave anechoic chamber. The experiments targeted the 1–7 GHz frequency band, the key working range for the system’s intended applications. As the second layer of the three-layer validation outlined in the Introduction, this experiment verifies the phase-control and beamforming algorithms under precisely known geometry: the compact 43.5 cm antenna spacing is deliberate, so that the measurement is dominated by the synchronization accuracy rather than by unknown propagation effects, complementing the link-stability test of Section 4.1 and the true spatial diversity exercised in Section 4.3.

**Figure 4:**
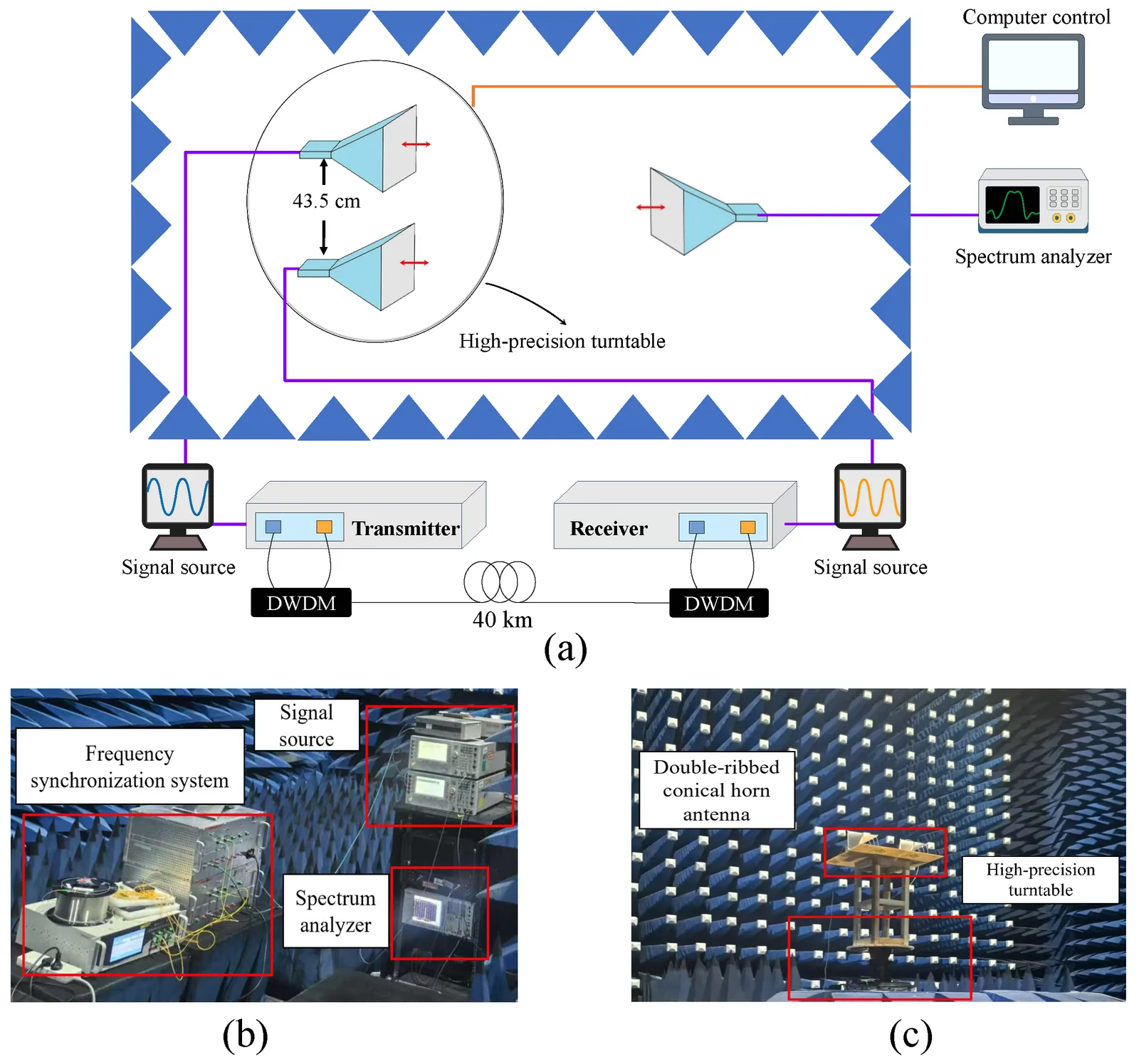
Experimental setup in the microwave anechoic chamber. (**a**) Overall schematic diagram of the experiment: two signal sources, each referenced by one end of the microwave-photonics frequency synchronization system (transmitter and receiver) interconnected by a 40 km fiber link, feed two horn antennas mounted on a high-precision turntable; the synthesized signal is received by the spectrum analyzer, and a computer control unit coordinates turntable rotation, signal-source settings, and data acquisition. (**b**) Photo of the experimental setup in the anechoic chamber, showing the physical arrangement of the signal sources, the synchronization system, and the spectrum analyzer. (**c**) Close-up of the antenna configuration: two transmitting antennas are mounted on the high-precision turntable with a fixed inter-element spacing of 43.5 cm and a rotation accuracy of 0.5°, which define the geometry used for the beam-scanning and beamforming measurements of Figure 5.

A testing system for coherent signal synthesis experiments is shown in Figure 4a. This system includes a high-precision turntable with two horn antennas mounted on it. It also features a frequency synchronization system with a transmitter and receiver to ensure consistent transmission and reception of signals. Two signal sources generate the signals, while a spectrum analyzer measures the received signals. These components work together to enable coherent operations such as constructive and destructive interference. The system also includes a computer control unit that automates tasks such as adjusting the turntable angle, setting signal source parameters, and acquiring and analyzing data from the spectrum analyzer. Figure 4b depicts the experimental setup within the microwave anechoic chamber, illustrating the arrangement of core components such as the signal source, spectrum analyzer, and synchronization system to clarify the interaction between components during testing. Figure 4c focuses on the antenna configuration: two transmitting antennas are mounted on a high-precision turntable with a fixed inter-element spacing of 43.5 cm, and an accuracy of 0.5°.

**Figure 5:**
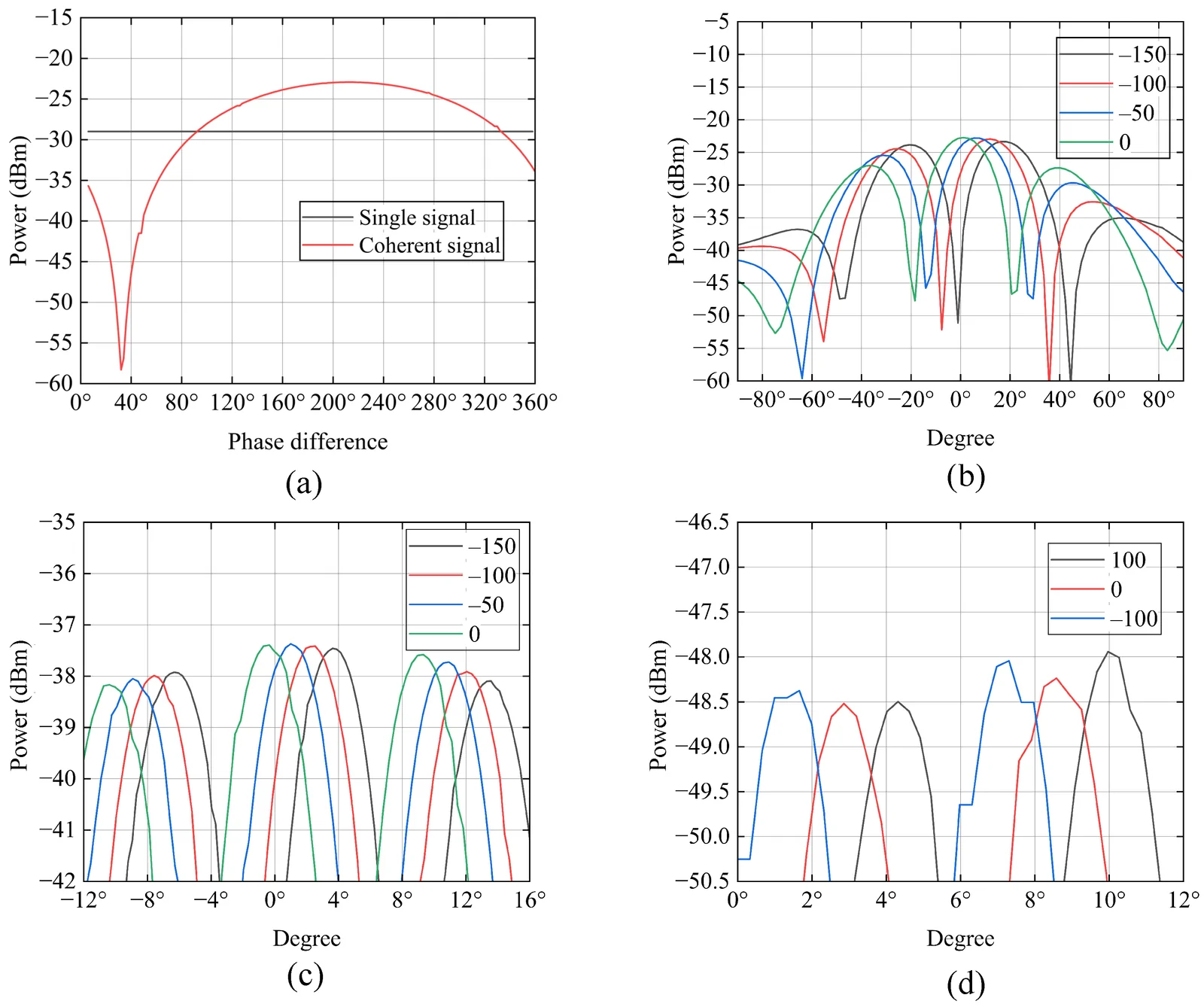
Beam-scanning and beamforming patterns in the anechoic chamber. (**a**) Beam-scanning pattern at 1 GHz: the horizontal axis is the relative phase difference between the two signal sources (°) and the vertical axis is the received power; the maximum intensity is obtained at a fixed phase difference of 200°, demonstrating phase coherence. (**b**–**d**) Beamforming patterns at 1, 4, and 7 GHz, respectively: the horizontal axis is the turntable rotation angle (°), the vertical axis is the received power, and each curve corresponds to a different applied phase shift (values in °); the beam-pointing angles follow the prediction of Equation (7) within the turntable resolution (0.5°).

To verify the system’s phase coherence, a phase scanning experiment is designed: the phase of the signal source in incremental steps is adjusted, the received signal intensity under each phase condition is measured, and corresponding phase scanning diagrams are plotted. This experiment aimed to confirm whether the system’s signals maintain coherence across phase variations—specifically, whether the maximum received signal intensity consistently concentrates at a specific phase angle. Figure 5a shows the phase scanning results at 1 GHz: the received signal intensity exhibited a periodic variation with scanning angle, and the maximum intensity was concentrated at a fixed phase angle across all measurements—clearly demonstrating good phase coherence. The images in the figure demonstrate that as the phase difference between the two signal sources changes, obvious coherent constructive and destructive interference phenomena emerge, with the maximum signal intensity detected at a phase difference of 200°. Although the two signal sources are synchronized via the frequency synchronization system, their initial phases have a slight mismatch—this discrepancy is mainly caused by minor hardware-related variations, such as inherent initial phase offsets of the PDRO in the signal sources or tiny asymmetries in the local clock distribution paths of the synchronization system. To align the phases of the two signals at the target position and thereby maximize the synthesized signal intensity, targeted phase adjustment of one signal source is necessary to offset this initial phase deviation.

To validate the beamforming capability of the distributed antenna system, the theoretical relationship between the inter-channel excitation phase difference and the resulting beam-steering angle must first be established. The theoretical relationship between the excitation phase difference between channels and the resulting beam-pointing angle is derived from the standard array theory expression in Equation (6):

(7) $$\theta_{0}=\arcsin\left(-\frac{\lambda }{2\pi d}\Delta \phi \right).$$

The negative sign indicates that the beam steers in the direction opposite to the channel with the phase advance. Throughout this work, positive azimuth is defined toward the channel that lags in phase, and the same convention is used in Equation (7) and in the measured patterns of Figure 5; with this convention, the predicted and measured signs agree. Based on this theory, calculations were performed for three representative frequency points: f=1 GHz, f=4 GHz, and f=7 GHz. With a fixed spacing of d=43.5 cm, a 1 GHz signal yields beam deflections of approximately −5.49°, −11.04°, and −16.70° for phase shifts of 50°, 100°, and 150°, respectively. The 4 GHz signal yields beam deflections of approximately −1.50°, −2.90°, and −4.32° for phase shifts of 50°, 100°, and 150°. In contrast, the 7 GHz signal yields beam deflections of approximately −1.5° and 1.32° for phase shifts of 100° and −100°, respectively. Experimental measurement results are shown in Figure 5b–d. The experimentally measured beam-pointing angles align closely with the theoretical predictions across all tested phase differences: the measured deviations from the predicted angles stay within the 0.5° resolution of the turntable in all cases. Furthermore, the observed steering direction consistently matches the sign relationship predicted by Equation (7).

The experimental results collectively validate one critical conclusion: the system’s beamforming and phase scanning functions are feasible across the 1–7 GHz band. All experiments confirmed that precise adjustments of phase and amplitude achieve stable directional beams and coherent signal synthesis. These findings provide a direct experimental basis for the design of future multi-band microwave systems based on the demonstrated distributed antenna architecture.

### 4.3. Experiment in the Field

**Figure 6:**
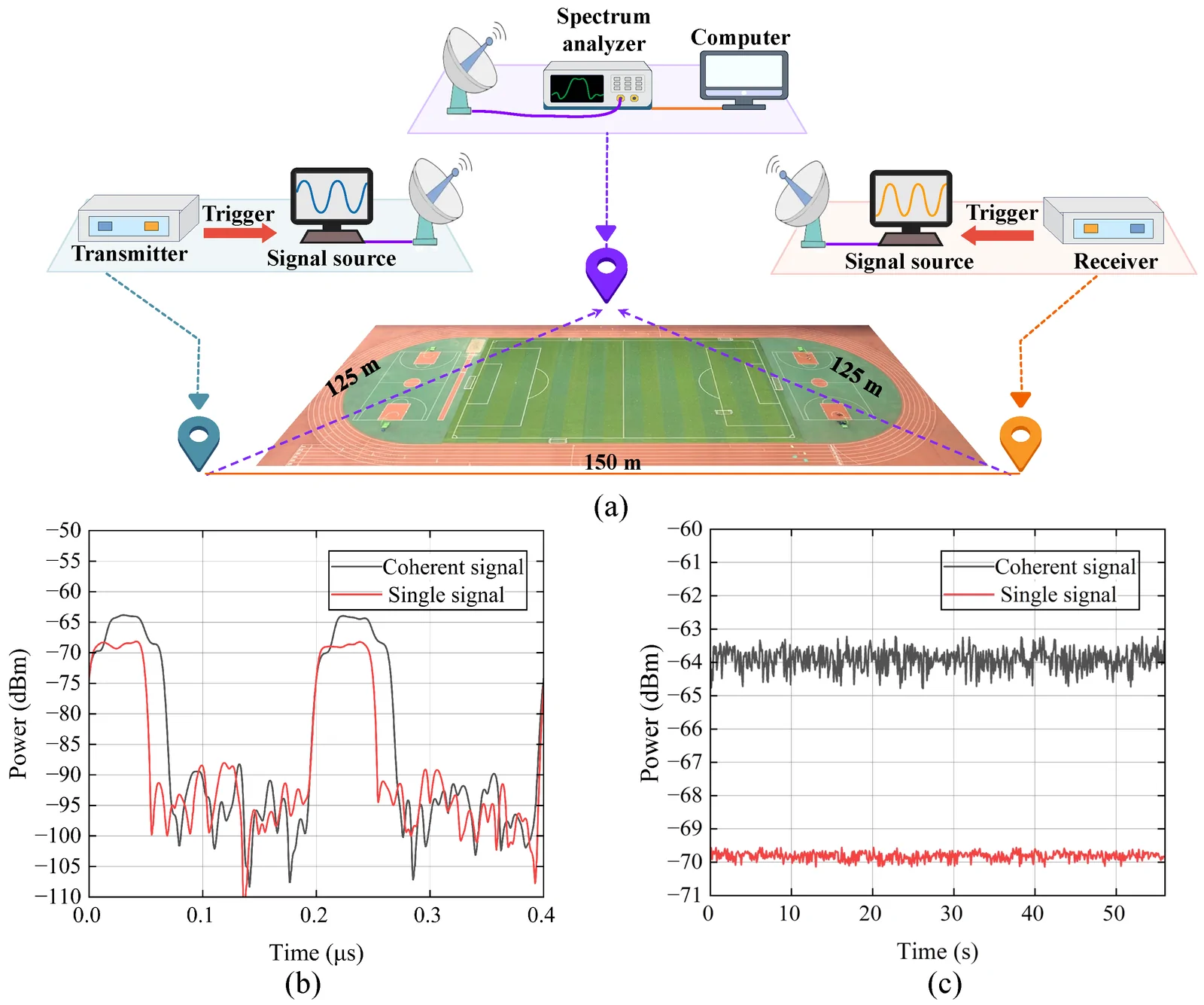
Field-test plan and stability of the synthesized signal. (**a**) Measurement plan of the field test. (**b**) Intensity stability of the continuous-wave coherent synthesis signal with and without synchronization. (**c**) Intensity stability of the pulse coherent synthesis signal with and without synchronization.

As the third layer of the validation logic, this field experiment verifies the third contribution: whether the synchronized sources can actually synthesize coherently under realistic spatial separation, as demanded by the application scenarios of distributed coherent radar and long-range communication. Previous frequency synchronization methods lack integrated high-precision signal source synchronization and comprehensive field validation, which limits their practical engineering applicability. To verify the signal synthesis stability and coherent gain performance of the distributed antenna system in real-world scenarios, we designed a field pulse signal synthesis experiment, as shown in Figure 6a. In this experiment, a 150 m free-space separation is set between two signal sources, with a frequency synchronization system placed at each end. These systems generate sinusoidal signals with amplitude and phase modulation, which are then transmitted through antennas. The signals are received by the antennas at the target point and processed by a spectrum analyzer.

First, before the experiment, we adjusted the output amplitude of the two signal sources. The core purpose of this operation was to ensure that the signal amplitudes reaching the spectrum analyzer were equal—if the amplitudes were inconsistent, any observed difference in synthesized signal strength could be attributed to amplitude deviation rather than the synchronization system’s performance. By calibrating the power output of each signal source, this non-synchronization-related error is eliminated, ensuring that subsequent gain measurements accurately reflect the effect of the microwave-photonics-based phase-locked loop. Second, the phase offset of one signal source was further adjusted until the amplitude of the synthesized signal reached its maximum. This step was critical because initial phase misalignment between the two sources would result in incomplete constructive interference, even with effective frequency synchronization. By optimizing the phase to achieve maximum initial synthesis amplitude, it was ensured that subsequent stability tests and gain measurements were based on the system’s optimal working state.

After completing equipment configuration, amplitude calibration, and phase optimization, the signal was received and measured. The results, presented in Figure 6b, show a 5.9 dB increase in amplitude, and the combined-signal amplitude fluctuation was maintained within 1 dB, meeting actual usage requirements. The signal sources were then set to pulse modulation mode and output pulse signals. To ensure temporal coherence of the two signals, the output time delay of the signal sources was adjusted until the pulses overlapped in time at the receiving end. The waveform image in Figure 6c compares the signal strength–time characteristics of a single signal source and the coherent signal of two sources: the synthesized signal shows a 5.9 dB increase in amplitude, with coherent gain loss maintained below 0.1 dB. This value is close to the theoretical coherent gain of dual-antenna systems, indicating that the synchronization system maintains high-efficiency coherent synthesis even in field environments.

This field experiment not only supplements the limitations of anechoic chamber experiments but also verifies the system’s adaptability to practical scenarios. The stable synthesis effect and 5.9 dB gain further confirm that the employed microwave-photonics-based phase-locked loop is a practical solution for distributed antenna systems.

We note two limitations of the present validation. First, the 40 km fiber used in the stability test was spooled in the laboratory; although this provides a controlled and repeatable environment for validating the dispersion-compensation and round-trip-cancellation chain, it does not fully reproduce the vibration and temperature environment of deployed inter-site fiber. A field campaign over deployed fiber is planned for future work. Second, the free-space separation in the field test (150 m) confirms the coherent-synthesis principle but does not yet exercise the full inter-site distances targeted by long-range distributed radar. These limitations bound the generality of the reported stability numbers and motivate the planned on-site experiments.

## 5. Conclusions

In summary, we report a system-level validation of coherent synthesis between two signal sources enabled by a microwave-photonics-based frequency synchronization system. The system was characterized through multi-stage experiments on a 40 km fiber link, in a microwave anechoic chamber, and in a 150 m field environment, demonstrating, in turn, the long-haul reference stability (Section 4.1), the broadband beamforming capability (Section 4.2), and the end-to-end coherent power combining (Section 4.3). Experimental results show specific precision: an $8.0\times 10^{-15}$ per second frequency stability was achieved over a 40 km fiber link; precise beamforming was verified in the anechoic chamber with a 5.9 dB enhancement in coherent gain; and stable signal synthesis was realized even with a 150 m free-space separation in field tests, meeting the precision requirements of distributed coherent systems.

Future research will further optimize the system’s hardware design and synchronization accuracy, and will extend the validation to deployed fiber links and to more than two apertures. In parallel, quantum time synchronization is being explored to calibrate the trigger time of radar signals: because coherent operation also requires the pulse arrival times at the target to be aligned, a time-domain calibration that does not rely on the microwave channel itself could further improve the joint time–frequency synchronization accuracy. More practical application scenarios will be explored to provide technical support for large-scale distributed radar systems and cooperative synthesis in communication systems.

## Figures and Tables

**Table 1:** Key parameters of the microwave-photonics frequency synchronization system.

Component	Parameter	Value
Laser diode (LD)	Wavelength (Tx/Rx)/linewidth	1553.33/1548.51 nm/100 kHz
Mach–Zehnder modulator (MZM)	Half-wave voltage/bandwidth	4 V/10 GHz
Photodetector (PD)	Bandwidth/responsivity	10 GHz/0.9 A/W
Bi-EDFA	Gain/noise figure	≥24 dB (per 80 km span)/3.7±0.2 dB
DCF	Compensated dispersion	80 km SMF equivalent per span
PDRO	Output frequencies/phase noise	$f_{1}=2.3$ GHz, $f_{2}=2.5$ GHz/−106 dBc/Hz at 100 Hz
OCXO	Frequency/stability	100 MHz/−135 dBc/Hz @100 Hz, −176 dBc/Hz at 100 kHz
PFD/LF/BPF/PS	Loop parameters	PFD phase noise −153 dBc/Hz at 10 kHz; third-order LF; PLL bandwidth 180 Hz

## Data Availability

The original contributions presented in this study are included in the article. Further inquiries can be directed to the corresponding author.
